# Selective Photokilling of Human Pancreatic Cancer Cells Using Cetuximab-Targeted Mesoporous Silica Nanoparticles for Delivery of Zinc Phthalocyanine

**DOI:** 10.3390/molecules23112749

**Published:** 2018-10-24

**Authors:** Özge Er, Suleyman Gokhan Colak, Kasim Ocakoglu, Mine Ince, Roger Bresolí-Obach, Margarita Mora, Maria Lluïsa Sagristá, Fatma Yurt, Santi Nonell

**Affiliations:** 1 Department of Nuclear Applications, Institute of Nuclear Science, Ege University, Bornova, Izmir 35100, Turkey; ozgeer88@gmail.com; 2 Advanced Technology Research & Application Center, Mersin University, Ciftlikkoy Campus, Yenisehir, Mersin 33343, Turkey; suleymangokhancolak@gmail.com; 3 Department of Energy Systems Engineering, Faculty of Technology, Tarsus University, Tarsus 33400, Turkey; kasim.ocakoglu@tarsus.edu.tr (K.O.); mine.ince@tarsus.edu.tr (M.I.); 4 Institut Quimic de Sarria, Universitat Ramon Llull, Via Augusta 390, 08017 Barcelona, Spain; rogerbresolio@iqs.url.edu; 5 Departament de Bioquímica i Biomedicina Molecular, Facultat de Biologia, Universitat de Barcelona, Avinguda Diagonal 645, E-08028 Barcelona, Spain; margarita.mora@ub.edu (M.M.); mlsagrista@ub.edu (M.L.S.)

**Keywords:** Zn(II) phthalocyanine, mesoporous silica nanoparticles, Cetuximab, singlet oxygen, photodynamic therapy

## Abstract

Background: Photodynamic therapy (PDT) is a non-invasive and innovative cancer therapy based on the photodynamic effect. In this study, we sought to determine the singlet oxygen production, intracellular uptake, and in vitro photodynamic therapy potential of Cetixumab-targeted, zinc(II) 2,3,9,10,16,17,23,24-octa(tert-butylphenoxy))phthalocyaninato(2-)-N^29^,N^30^,N^31^,N^32^ (ZnPcOBP)-loaded mesoporous silica nanoparticles against pancreatic cancer cells. Results: The quantum yield (*Φ*_Δ_) value of ZnPcOBP was found to be 0.60 in toluene. In vitro cellular studies were performed to determine the dark- and phototoxicity of samples with various concentrations of ZnPcOBP by using pancreatic cells (AsPC-1, PANC-1 and MIA PaCa-2) and 20, 30, and 40 J/cm^2^ light fluences. No dark toxicity was observed for any sample in any cell line. ZnPcOBP alone showed a modest photodynamic activity. However, when incorporated in silica nanoparticles, it showed a relatively high phototoxic effect, which was further enhanced by Cetuximab, a monoclonal antibody that targets the Epidermal Growth Factor Receptor (EGFR). The cell-line dependent photokilling observed correlates well with EGFR expression levels in these cells. Conclusions: Imidazole-capped Cetuximab-targeted mesoporous silica nanoparticles are excellent vehicles for the selective delivery of ZnPcOBP to pancreatic cancer cells expressing the EGFR receptor. The novel nanosystem appears to be a suitable agent for photodynamic therapy of pancreatic tumors.

## 1. Introduction

Pancreatic cancer is the 13th most common cancer and the 4–8th leading cause of cancer deaths worldwide. It occurs in the majority of cases with early locoregional spread and distant metastases [1,2]. The lack of symptoms in early stages causes late diagnoses, which detracts from the effectiveness of treatments such as surgery, radiotherapy, or chemotherapy [1,3]. Before 2008, the only potential curative treatment offered to patients was a combination of surgery and adjuvant chemotherapy, with a five-year survival rate near to 40%. Targeted radionuclide therapies, using radiolabeled peptides capable of binding to receptors overexpressed by cancer cells or radiolabeled antibodies for tumor-specific antigens, and therapies directed against the Epidermal Growth Factor Receptor (EGFR) [4,5] have been proposed as viable alternatives to chemotherapy and external beam radiation therapies. Targeting agents for EGFR include tyrosine kinase inhibitors and monoclonal antibodies such as Cetuximab (C225). 

Photodynamic therapy (PDT) is a treatment modality that combines light, a drug acting as a photosensitizer (PS) and tissue oxygen, all innocuous for the cells by themselves, to eliminate tumors [6]. It can be used for the treatment of various ophthalmic, dermatologic, cardiovascular, and predominantly, oncologic diseases. The light-activated PSs create highly reactive oxygen species such as singlet oxygen (^1^O_2_), which destroy cells in the tissue [7,8]. Phthalocyanines (Pcs) show strong potential as PSs owing to their strong absorption in the far visible region of the solar spectrum (between 640 and 700 nm), which matches the ‘therapeutic window’ (600–800 nm). Moreover, they are chemically, photochemically, and thermally stable and present high efficiency of ^1^O_2_ generation [9,10,11,12,13]. However, many Pcs are hydrophobic and little soluble in aqueous medium, where they tend to aggregate, which weakens their photophysical properties and ^1^O_2_ production ability [14]. The use of delivery vehicles such as nanoparticles (NPs), has emerged as a valuable option to overcome these limitations [15,16]. In addition, NPs have the capability to selectively accumulate in the malignant tissue, either passively, by the enhanced permeability and retention (EPR) effect [17], or actively, if their surface is decorated with ligands that can be selectively recognized by specific markers of the tumor cells [18,19]. Thus, these targeted drug delivery systems are able to modify the pharmacokinetics and biodistribution of their associated drugs. Indeed, nanomedicine is starting to make an impact in areas such as disease imaging diagnosis and drug delivery [20,21]. 

In recent years, the use of NPs in PDT has increased dramatically due to both the ability to deliver a large number of PS molecules to a single cell and the enhanced targeting potential offered by surface decoration with suitable ligands, which may significantly reduce the systemic toxicity associated with classical PDT therapy [21,22,23]. Although several nanomaterials have been explored for nanomedicine [16], silica nanoparticles, particularly of the mesoporous type (MSNPs), appear of great interest for PDT due to their easy preparation, wide range of sizes, biocompatibility, high drug loading capacity, internalization by a large number of cell lines, chemical inertness, large surface area, and easy surface decoration with ligands and solubilizing agents [24,25,26]. Of specific interest for this work, MSNPs decorated with EGFR-targeting monoclonal antibody Cetuximab have been used for the delivery of doxorubicin and gefitinib to PC9-DR cells and tumors [27]. Thus, the aim of this study has been to assess the potential of MSNPs decorated with Cetuximab for the photodynamic killing of pancreatic cancer cells.

## 2. Results

### 2.1. Synthesis of Zinc(II) 2,3,9,10,16,17,23,24-octa(tert-butylphenoxy))phthalocyaninato(2-)-N^29^,N^30^,N^31^,N^32^ (ZnPcOBP)

**Scheme 1:**
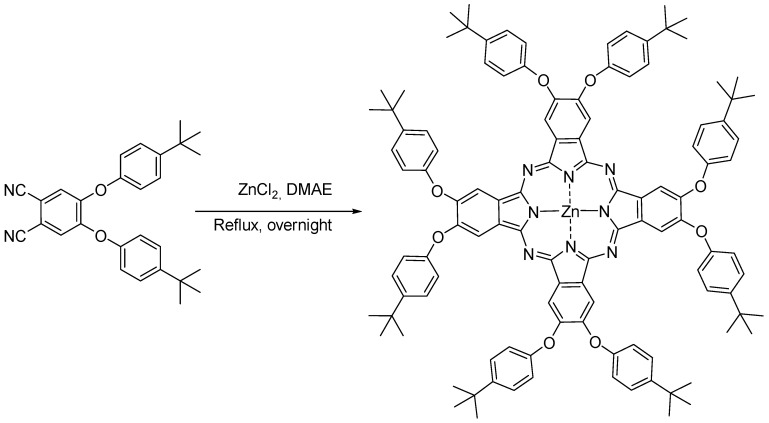
Synthesis of Zinc (II) 2,3,9,10,16,17,23,24-octa(*tert*-butylphenoxy)) phthalocyaninato(2-)-N^29^,N^30^,N^31^,N^32^ (ZnPcOBP).

ZnPcOBP was synthesized in 23% yield by means of the cyclotetramerization of 4,5-di(*p-tert*-butylphenoxy)phthalonitrile in the presence of ZnCl_2_ in dimethylaminoethanol (DMAE; Scheme 1). The spectroscopic data are in agreement with published data [28]. 

### 2.2. Preparation of Nanoparticles

**Scheme 2:**
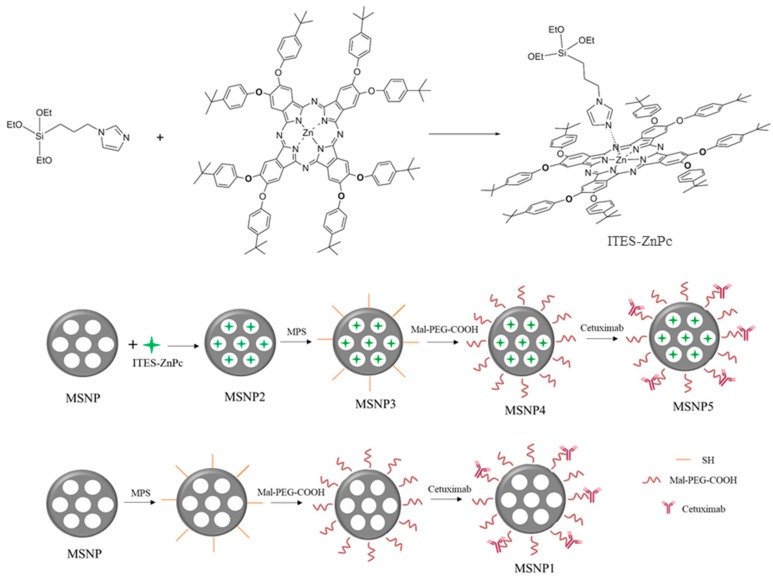
Preparation of the nanoparticles.

The preparation of nanoparticles was carried out in five steps (Scheme 2): (i) preparation of the complex between ZnPcOBP and 3-(2-imidazol-1-yl)propyltriethoxysilane (ITES-ZnPc); (ii) preparation of mesoporous silica nanoparticles with entrapped ZnPcOBP (MSNP2); (iii) modification of the nanoparticle surface with mercaptopropyl groups (MSNP3); (iv) anchoring of polyethyleneglycol chains (PEG; MSNP4); (v) anchoring of cetuximab (MSNP5). A phthalocyanine-free nanoparticle analog to MSNP5 was prepared as control (MSNP1).

### 2.3. Characterization of Nanoparticles

The size and zeta potential of the nanoparticles were measured by dynamic light scattering (DLS). The results are shown in Table 1. ZnPcOBP-containing nanoparticles were larger than phthalocyanine-free nanoparticles due to the grafting of ITES, the increase in size and concomitant changes in the zeta potential after each preparation step being a good indication that the sequential surface modifications had been successfully accomplished. 

Field emission-scanning electron microscopy (FE-SEM) and transmission electron microscopy (TEM) were used to obtain images of the nanoparticles (Figures S1 and S2 respectively). The size of the MSNP3 nanoparticles was determined to be 60 ± 10 nm by analysis of the FE-SEM images. In the TEM images, the porous structure of MSNP3 can be clearly seen (Figure S2). In addition, energy-dispersive X-ray spectroscopy (EDS, Figure S3) confirmed the presence of Si, O and S, whereby the detected S element represents -SH groups on the surface of the nanoparticle.

The IR spectra of ZnPcOBP, ITES, the complex ITES-ZnPc, and the nanoparticles MSNP3, MSNP4, and MSNP5 are shown in Figure S4. Comparison of the spectra for MSNP4 and MSNP5 show that cetuximab is attached to the nanoparticles in MSNP5 as indicated by the appearance of two new bands (1641 and 1534 cm^−1^), assigned to the normal modes of vibration of amide I and amide II of the antibody backbone, respectively [29]. 

**Figure 1:**
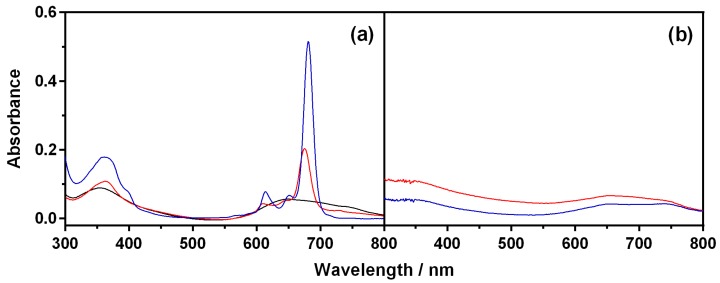
(**a**) Absorbance spectrum of ZnPcOBP in toluene (blue), acetonitrile (red), and methanol (black); (**b**) Absorbance spectra of MSNP3 (blue) and MSNP4 (red) in methanol.

UV-VIS spectra of ZnPcOBP in toluene, acetonitrile, and methanol are shown in Figure 1a. ZnPcOBP is in monomeric form in toluene, as deduced from the presence of two sharp peaks at 370 nm and 680 nm. However, it is slightly aggregated in acetonitrile and extensively aggregated in the more polar solvent methanol, as indicated by the decrease in absorbance and broadening of the bands. The UV-VIS absorbance spectra of MSNP3 and MSNP4 (1 mg) dispersed in methanol are shown in Figure 1b. It is worth noting that when the suspensions were centrifuged, no ZnPcOBP could be found in the supernatant. The similarity with the spectrum of ZnPcOBP in methanol indicates that the phthalocyanine is extensively aggregated in the NPs. 

The amount of ZnPcOBP in the nanoparticles was determined by extracting it with THF, measuring its absorbance at 680 nm, and interpolating in a calibration curve (ZnPcOBP in THF in the range 0.1–1 μM; Figure S5). Thus, it was observed that 1 mg MSNP3 contains 10.4 μg ZnPcOBP (Figure S6), 1 mg MSNP4 contains 6.2 μg ZnPcOBP (Figure S7), and 1 mg MSNP5 contains 3.8 μg ZnPcOBP (Figure S8), consistent with the increase of the nanoparticle mass upon sequential addition of surface components.

### 2.4. Singlet Oxygen Production 

**Figure 2:**
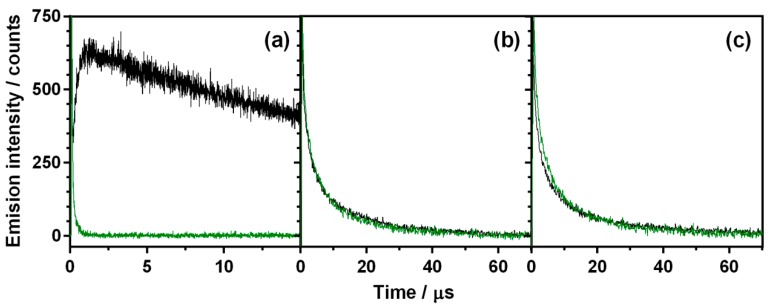
Time-resolved phosphorescence intensity 1275 nm (black) and 1110 nm (green) upon 355 nm pulsed laser excitation of (**a**) ZnPcOBP in toluene; (**b**) MSNP3 in methanol; (**c**) MSNP4 in methanol.

When exposed to light, many PSs kill cancer cells by producing singlet oxygen. We therefore assessed the ability of ZnPcOBP to photosensitize the production of this reactive oxygen species. Thus, small amounts of a concentrated stock solution of ZnPcOBP in toluene were diluted into 2 mL toluene and the time-dependent change of phosphorescence intensity upon pulsed laser excitation was recorded at 1275 nm (where singlet oxygen emits) and at 1110 nm (where the triplet ZnPcOBP emits). Unequivocal ^1^O_2_ production was thus demonstrated by the observation of a long-lived rise-and decay signal at 1275 nm, while at 1110 nm only a short-lived decay signal could be observed (Figure 2a). 

The quantum yield of singlet oxygen production (*Φ*_Δ_; Figure S9) was determined by comparing the 1275 nm phosphorescence intensity of ZnPcOBP with that of a reference photosensitiser, 1*H*-phenalen-1-one (PN), for which *Φ*_∆_ = 0.92 [30,31,32]. We found that the *Φ*_Δ_ value of ZnPcOBP in toluene is 0.60, in good agreement with the value of 0.73 reported by Maree and Nyokong [28] in chloroform. 

The production of singlet oxygen by the nanoparticles was likewise investigated in methanol suspensions. For this study, 1 mg of MSNP3 and 1 mg of MSNP4 were dispersed in 1 mL methanol and the phosphorescence was recorded at 1275 and 1110 nm. As shown in Figure 2b,c, the two traces are indistinguishable, which indicates that MSNP3 and MSNP4 do not release any detectable amount of singlet oxygen. As an additional control, the experiments were repeated in the presence of sodium azide (NaN_3_), a well-known singlet oxygen quencher [33]. Again, the signals did not change, which confirms that production of singlet oxygen by the nanoparticles is negligible (Figure S10).

### 2.5. Release of ZnPcOBP from the Nanoparticles

**Figure 3:**
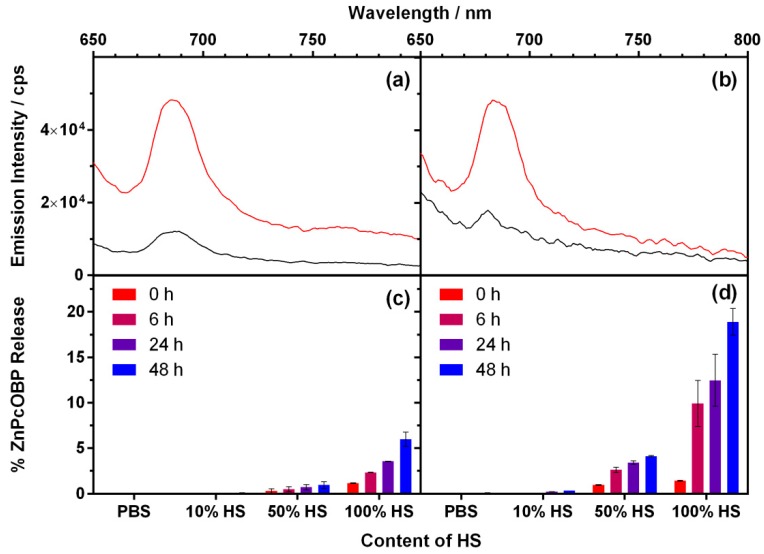
Fluorescence spectra of the supernatant after centrifugation of (**a**) MSNP4 and (**b**) MSNP5 nanoparticle suspensions incubated overnight in the presence (red) and absence (black) of BSA. Influence of human serum (HS) content on the release of ZnPcOBP from (**c**) MSNP4 and (**d**) MSNP5 nanoparticles as a function of the incubation time in PBS-HS mixtures.

We observed that ZnPcOBP could be quantitatively extracted from the nanoparticles by using non-polar solvents, such as THF, or coordinating solvents, such as pyridine (Figure S11). We therefore hypothesized that proteins, widely available in biological media, might be able to extract ZnPcOBP from the nanoparticles facilitating their delivery to the cancer cells. To test this hypothesis, 2 mg of MSNP4 or MSNP5 and 250 μL of bovine serum albumin (BSA) 150 μM were dispersed in 2 mL of phosphate-buffered saline (PBS; pH 7.4) and the mixtures allowed to equilibrate overnight under gentle magnetic stirring. The suspensions were then centrifuged and the fluorescence of the supernatants was recorded. Nanoparticle suspensions without BSA were used as controls. The characteristic fluorescence bands of ZnPcOBP were remarkably more intense in the samples containing BSA (Figure 3a,b), confirming that ZnPcOBP can be released from the nanoparticles in the presence of proteins. 

Since cell incubation media usually contain 10% fetal bovine (or calf) serum, we investigated the release of ZnPcOBP from the nanoparticles in the presence of human serum (HS). Figure 3c,d shows that the release, measured by fluorescence of the supernatant after centrifugation, is negligible under typical incubation conditions (PBS containing 10% human serum incubated for up to 48 h). Increasing the concentration of human serum and the incubation time led to concomitantly higher release efficiencies. 

### 2.6. Cellular Uptake of MSNP4 and MSNP5

**Figure 4:**
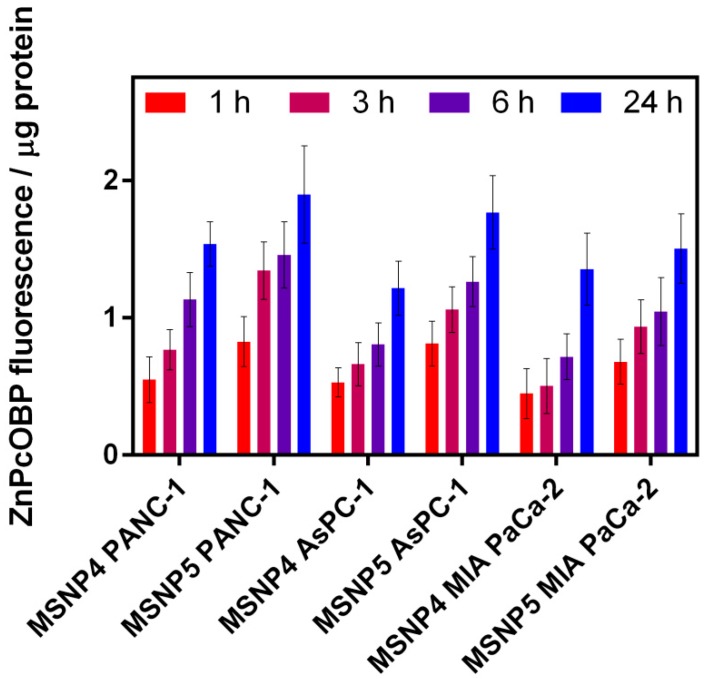
Cellular uptake kinetics of MSNP4 and MSNP5 nanoparticles on PANC-1, AsPC-1 and MIA PaCa-2 cells.

We studied the cellular uptake for MSNP4 and MSNP5 nanoparticles on the pancreatic tumor cell lines PANC-1, AsPC-1, and MIA PaCa-2. AsPC-1 and PANC-1 cells express more EGFR than MIA PaCa-2 cells [34]. Thus, appropriate amounts of the nanoparticles were added to the cells to deliver 1 µM ZnPcOBP and the extent of uptake was assessed at four incubation times (1 h, 3 h, 6 h and 24 h). The results are summarized in Figure 4. 

A consistent increase in ZnPcOBP fluorescence per microgram of cellular protein was observed upon increasing the incubation time for all cell lines. For any given cell line, nanoparticle uptake was higher for MSNP5 than for MSNP4, consistent with the presence and absence, respectively, of Cetuximab on their surface. The enhancement was cell dependent, namely 10%, 23%, and 46% higher in MIA-PaCa-2, PANC-1, and AsPC-1 cells, respectively, in agreement with the different expression levels of EGFR in these cells [34]. In absolute terms, the highest cellular uptake of MSNP5 nanoparticles was observed in PANC-1 cells, followed by AsPC-1 and MIA PaCa-2 cells.

### 2.7. Cell Photocytotoxicity Studies

**Figure 5:**
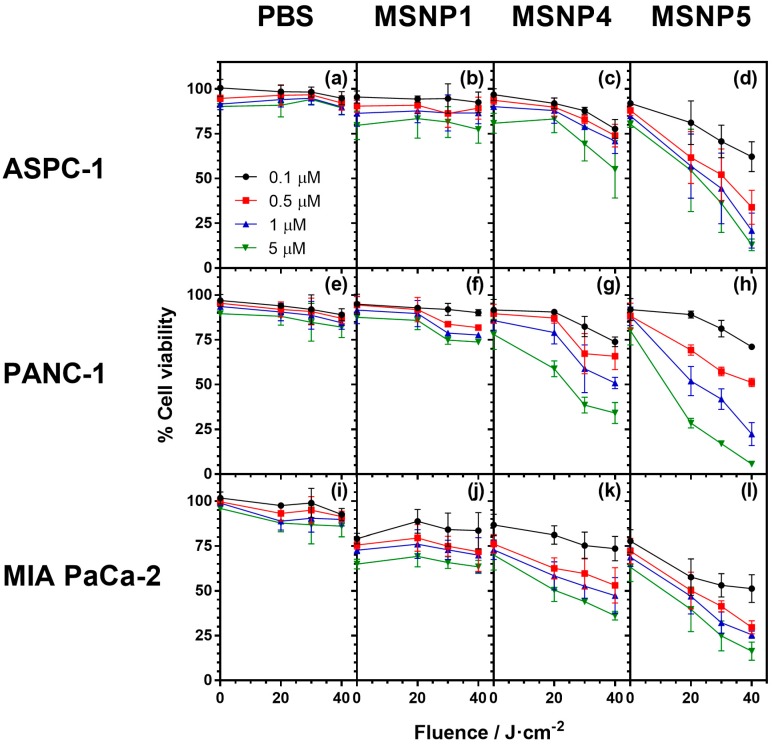
ASPC-1 (**a**–**d**), PANC-1 (**e**–**h**), and MIA PaCa-2 (**i**–**l**) cell viability (expressed as percentages) after incubation with: PBS (**a**,**e**,**i**), MSNP1 (**b**,**f**,**j**), MSNP4 (**c**,**g**,**k**) or MSNP5 (**d**,**h**,**l**) nanoparticles suspended in PBS, at various ZnPcOBP concentrations and light fluences (20, 30, and 40 J/cm^2^). In the panels (**a**,**e**,**i**), equivalent amounts of PBS to those needed to reach the indicated concentrations of ZnPcOBP were added in the wells to assess the effect of medium dilution with PBS. ZnPcOBP concentrations are 0.1 μM (black), 0.5 μM (red), 1.0 μM (blue), and 5 μM (green). Cells incubated with complete growth medium, to which 100% viability was assigned, were used as controls to calculate the cell viability of the cells treated at the different conditions. Data are the mean ± SD from three independent experiments.

In vitro cell photocytotoxicity studies were carried out by using PANC-1, AsPC-1, and MIA PaCa-2 cell lines. Water-insoluble ZnPcOBP and MSNP3 nanoparticles were first dissolved or suspended, respectively, in dimethylacetamide (DMA) and then added to the incubation medium of the cells. MSNP1, MSNP4, and MSNP5 nanoparticles were delivered suspended in PBS. Cells were incubated in the dark for 24 h with various concentrations of free ZnPcOBP or the nanoparticles and then exposed to various fluences of LED red light or kept in the dark for control purposes. Finally, cell viability was assessed 24h after the treatment. Figure 5 shows the photocytotoxicity results after the different treatments. 

Experiments carried out by adding the PS or NPs in DMA revealed that ZnPcOBP alone had only a modest photodynamic activity. Moreover, DMA exerted a substantial dark toxicity by itself and enhanced the dark- and phototoxicity of the MSNP3 nanoparticles, preventing any sound comparison with the results of the other nanosystems (Figure S12). On the contrary, the dark toxicity of the nanosystems delivered in PBS was negligible (Figure 5a,e,i). 

MSNP1 nanoparticles, which contain no ZnPcOBP, showed almost no dark toxicity for the PANC-1 and AsPC-1 cells, but cell viability decreased in a concentration-dependent manner for the MIA-PaCa-2 cells down to 60% at the maximum concentration (Figure 5b,f,j). The dark toxicity of the other nanosystems (MSNP4 and MSNP5) was very similar to that of MSNP1, ruling out any important contribution of the ZnPcOBP or Cetuximab. PANC-1 and AsPC-1 cells viability in the dark, after their incubation with ZnPcOBP-loaded-MSNP4 (coated with PEG; Figure 5c,g,k) and -MSNP5 (coated with PEG and Cetuximab; Figure 5d,h,l) nanoparticles, was near to 80% for the higher photosensitizer dose, the MIA PaCa-2 cells again being more sensitive with a cell viability of 70% at the higher photosensitizer dose. 

Upon irradiation, a decrease in cell viability was observed that was concentration and light- fluence dependent. Under the same experimental conditions, cell viability was lower when cells were incubated with MSNP5 nanoparticles than with MSNP4 nanoparticles. Thus, at 5.0 μM ZnPcOBP and a light fluence of 40 J/cm^2^, the cell viability was 35%, 55%, and 39% for the PANC-1, AsPC-1, and MIA PaCa-2 cells incubated with MSNP4, respectively, while the corresponding values for MSNP5 were 6.2%, 12.5%, and 17.5%. Again, these results are in agreement with the cell uptake and EGFR expression level in these cells.

## 3. Discussion

The results above highlight the potential of MSNPs coated with Cetuximab for the photodynamic killing of pancreatic cancer cells. Mesoporous silica nanoparticles have been engineered for the selective delivery of ZnPcOBP to the cells. The size of the nanoparticles (260–300 nm) is adequate for photodynamic applications and their slightly-negative zeta potential precludes their aggregation into larger structures.

The coordination ability of its central Zn cation allows reversible binding of the phthalocyanine to nanoparticles with imidazoles grafted onto their surface. This kind of binding facilitates the release into the cancer cells. Indeed, it was found that ZnPcOBP could be extracted from the nanoparticles using suitable solvents, as well as BSA and serum proteins. While in principle this might lead to a premature release in the presence of blood proteins, it should not be difficult to address this issue at the pharmacology stage, modifying the nanoparticle structure and binding affinity.

Regarding its photosensitizing properties, ZnPcOBP is an excellent ^1^O_2_ PS with a quantum yield of *Φ*_∆_ = 0.6 in the monomeric form. However, it does not produce ^1^O_2_ when bound to the nanoparticles because it is extensively aggregated (see Figure 1) and it is well known that aggregation suppresses the ability of phthalocyanines to produce ^1^O_2_ [35]. ZnPcOBP aggregation may prove advantageous to avoid unwanted photo-induced damage to other components in its pharmaceutical formulations.

When delivered in DMA solutions, ZnPcOBP showed only a small photodynamic activity against cancer cells, in contrast with the results for its nanoparticle-bound formulations. Indeed, MSNP3 nanoparticles show quite high phototoxic effects against AsPC-1, PANC-1, and MIA PaCa-2 cell cultures; however, this is probably due to the DMA solvent needed to solubilize them, which makes them also quite toxic in the dark. Their phototoxicity might be due, at least in part, to extraction of ZnPcOBP by DMA and/or cell membrane disruption by DMA favoring nanoparticle internalization.

In contrast, MSNP4 and MSNP5 are dispersible in PBS and show phototoxicity on AsPC-1, PANC-1, and MIA PaCa-2 cells, MSNP5 nanoparticles being superior to MSNP4. This is due to the presence of the Cetuximab antibody on their surface that favors cell recognition and receptor-mediated cell internalization, which, in turn, allows the release of ZnPcOBP within the cells. In vitro photodynamic results are consistent with cellular uptake and largely reflect the different levels of EGFR expression on the three cell lines. Therefore, the results presented clearly suggest that nanoparticles coated with PEG and cetuximab (MSNP5) could be an efficient vehicle for delivery of the photosensitizer ZnPcOBP to tumoral pancreatic cells with high EGFR expression in a targeted fashion.

## 4. Materials and Methods

### 4.1. Materials

Triethoxy-3-(2-imidazolin-1-yl)propylsilane (ITES), hexadecyl trimethyl ammonium chloride (CTAC), triethylamine (TEA), tetraethyl orthosilicate (TEOS), (3-mercaptopropyl) trimethoxysilane (MPS), Mal-PEG-COOH, 1-ethyl-3 (3-dimethylaminopropyl) carbodimide (EDC) were purchased from Sigma-Aldrich (Saint Louis, MO, USA). Cetuximab (Erlotinib) was supplied from Merck Serono (Darmstadt, Germany). The chemicals used in the in vitro studies were supplied from CLS-Cell Line Service. The other chemicals were purchased from Merck (Darmstadt, Germany). 4,5-Di(*p-tert*-butylphenoxy)phthalonitrile was synthesized as described previously [36].

### 4.2. Synthesis and Derivatitzation of Mesoporous Silica Nanoparticles

#### 4.2.1. Synthesis of Zinc (II) 2,3,9,10,16,17,23,24-octa(tert-butylphenoxy))phthalocyaninato (2-)-N^29^,N^30^,N^31^,N^32^ (ZnPcOBP)

A mixture of 4,5-di(*p-tert*-butylphenoxy)phthalonitrile (300 mg, 0.7 mmol) and ZnCl_2_ (53 mg, 0.28 mmol) in dimethylaminoethanol (DMAE; 5 mL) was heated at reflux overnight with stirring under argon atmosphere. After cooling to room temperature, the solvent was removed and the residue was washed with a MeOH/H_2_O (5:1) mixture. The crude product was purified by column chromatography on silica gel, (DCM/THF, 10:1) as eluent to yield ZnPcOBP (80 mg, 0.04 mmol) as a green solid. Yield: 23%.

^1^H-NMR (400 MHz, CDCl_3_): δ (ppm) = 8.93–8.45 (m, 8H), 7.40 (d, 4H, *J* =8 Hz), 7.3–7.2 (m, 12H), 7.1–7.0 (m 12H), 6.8 (d, 4H, *J* = 8 Hz), 1.32–1.29 (m, 72H). IR (ATR): ν (cm^−1^) = 3647, 2955, 1719, 1602, 1506, 1487, 1446, 1362, 1291, 1178, 890, 720. UV-Vis (CHCl_3_): λ_max_ (log ε) = 676 (1.46), 646 (2.05), 608 (2.23), 358 (4.51). MS (MALDI): *m*/*z*: 1763.205 [M^+^].

#### 4.2.2. Preparation of ITES-ZnPc Complex

The ITES-ZnPc complex was prepared by combining of ZnPcOBP and ITES at the molar ratio of 1:10 following the study of Durgun et al. [37]. ZnPcOBP (0.011 mmol) was dissolved in 2 mL of absolute ethanol under magnetic stirring. Then, 0.11 mmol ITES was added to the mixer and stirred for 24 h in the dark and at room temperature. After 24 h, the ITES-ZnPc core was collected and used without further purification.

#### 4.2.3. Synthesis of Mesoporous Silica Nanoparticles and Encapsulation of ZnPcOBP (MSNP2)

The mesoporous silica nanoparticles were synthesized based on the method used by Chen et al. [38]. A quantity of 2.0 g of CTAC (6.2 mmol) and 20 mg of triethylamine (TEA; 0.2 mmol) were dissolved in 30 mL of bidistilled water and mixed at room temperature for 1 h. Then, the previously prepared ITES-ZnPc complex was added to the mixture and stirred for an additional hour. Afterwards, 2.0 mL of TEOS (9.0 mmol) was added rapidly and the resulting mixture was stirred at 95 °C for 1 h. At the end of the reaction, the mixture was allowed to cool and centrifuged to collect (10 min, 3000 rpm, room temperature). The collected product (MSNP2) was then washed with water and ethanol to remove residual reagents. Then, the product was extracted three times with 1 wt% NaCl solution in methanol for 3 h to remove the CTAC. 

#### 4.2.4. Synthesis of MSNP3

MSNP2 nanoparticles were functionalized with -SH groups as described in ref. [38]. All of the synthesized MSNP2 nanoparticles were dispersed in 20 mL absolute ethanol and 2.0 mL of (3-mercaptopropyl)trimethoxysilane (MPS; 11 mmol) were added. The mixture was closed and kept in the dark under stirring for 12–24 h at 86–90 °C. Afterwards, the reaction mixture was cooled down and centrifuged (10 min, 3000 rpm, room temperature). The obtained nanoparticles (MSNP3) were washed several times with ethanol to remove the residual MPS.

#### 4.2.5. Synthesis of MSNP4

MSNP3 nanoparticles were PEGylated with Mal-PEG-COOH chain as described by Karra et al. [39]. MSNP3 nanoparticles were dispersed in water (pH 6.5–7) in a round bottom flask. Then the same amount of Mal-PEG-COOH was added to the flask (1:1 wt ratio of MSNP3:Mal-PEG-COOH) and stirred for 24 h at room temperature. Later, MSNP4 were collected from the flask, centrifuged and washed with water for 3 times (10 min, 3000 rpm, room temperature).

#### 4.2.6. Synthesis of MSNP5

Conjugation of Cetuximab to the surface of the MSNP4 nanoparticles was performed according to Wang et al. [19]. MSNP4 nanoparticles were dispersed in purified water and the pH was adjusted to 4.8. Then EDC (5:2 wt ratio of MSNP4:EDC) was added and the mixture was left under stirring at room temperature for 30 min. Afterwards, the carboxyl-activated MSNP4 nanoparticles were centrifuged and washed twice with PBS. Next, the activated NPs and Cetuximab (5:2 wt ratio of activated NPs:Cetuximab) were allowed to react overnight in PBS at room temperature. Finally, MSNP5 were centrifuged and washed with PBS. 

#### 4.2.7. Synthesis of MSNP1

MSNP1 nanoparticles were prepared following the same procedure as for MSNP5 without adding ITES-ZnPc. 

#### 4.2.8. Nanoparticle Characterization

The size and morphology of the synthetized NPs were characterized using: (i) field emission scanning electron microscopy (FE-SEM; Zeiss/Supra 55, Carl Zeiss AG, Oberkochen, Germany), (ii) transmission electron microscopy (TEM; JEOL JEM-2100 (UHR), Jeol Ltd., Tokyo, Japan) and (iii) dynamic light scattering (DLS; Zetasizer-Malvern Nano ZS90, Malvern Instruments Ltd., Worcestershire, UK) techniques. ATR-infrared spectra of the NPs were recorded in a Spectrum Two FTIR-ATR (Perkin Elmer, Waltham, MA, USA).

### 4.3. Spectroscopic Techniques

UV-Vis absorption spectra of the samples in various solvents were recorded with a Varian Cary 6000i spectrophotometer (Palo Alto, CA, USA). Fluorescence spectra of the samples were measured with a Fluoromax-4 spectrofluorometer (Horiba Jobin-Ybon, Edison, NJ, USA).

Production of ^1^O_2_ was studied by time-resolved near-infrared phosphorescence using a setup described in detail elsewhere [40,41]. Briefly, a pulsed Nd:YAG laser (FTSS355-Q, Crystal Laser, Berlin, Germany) working at 355 nm (third harmonic; 0.5 μJ per pulse; 1 kHz repetition rate) was used for sample excitation. A 1064 nm rugate notch filter and an uncoated SKG-5 filter were placed at the exit port of the laser to remove any residual component of its fundamental emission in the near-infrared region. The luminescence exiting from the sample was filtered by a 1100 nm long-pass filter and a narrow bandpass filter at 1275 nm. A thermoelectric-cooled near-infrared sensitive photomultiplier tube assembly (H9170-45, Hamamatsu Photonics, Hamamatsu, Japan) was used as detector. Photon counting was achieved with a multichannel scaler (NanoHarp 250, PicoQuant, Berlin, Germany).

The kinetic parameters governing the production and decay of ^1^O_2_ were determined by fitting the model below to the time-resolved phosphorescence intensity at 1275 nm (Equation (1)):

*S*(t) = *S*(0) × *τ*_∆_/(*τ*_∆_ − *τ*_T_) × [exp(−t/*τ*_∆_) − exp(−t/*τ*_T_)] (1)

where *τ*_∆_ and *τ*_T_ are ^1^O_2_ and triplet ZnPcOBP lifetimes, and the pre-exponential factor *S*(0) is an instrumental quantity proportional to the ^1^O_2_ production quantum yield (*Φ*_Δ_). The *Φ*_Δ_ value for ZnPcOBP was determined by comparison of its *S*(0) value to that of an optically-matched solution of the reference PS phenalenone in toluene, for which *Φ*_Δ_ = 0.92 [30,31,32].

### 4.4. Cellular Techniques

#### 4.4.1. Cell Lines and Culture Conditions

Cell culture studies were carried out using AsPC-1 (human pancreatic adenocarcinoma), PANC-1 (human pancreas/duct epithelioid carcinoma), and MIA PaCa-2 (human pancreas carcinoma) cell lines. AsPC-1 cells were cultured in RPMI 1640 containing 10% heat-inactivated FBS, 1% l-glutamine, and 1% penicillin-streptomycin. PANC-1 and MIA PaCa-2 cells were cultured in DMEM containing 10% heat-inactivated FBS, 1% l-glutamine, and 1% penicillin-streptomycin. All the cells were maintained at 37 °C in an incubator containing 5% CO_2_.

#### 4.4.2. Cellular uptake Assays

A quantity of 1.7 × 10^4^ cells was added to 96 wells plate and incubated at 37 °C for 24 h to achieve an 80% confluence. Afterwards, the culture medium was removed and 1 µM photosensitizer (alone or adsorbed onto NPs) with fresh medium was added. After the corresponding incubation times, the compounds were removed, the wells were washed 3 times with PBS. Then 100 µL of SDS (4%) were added and incubated 30 min more at 37 °C. Then 100 µL DMA was added and incubated for 30 min more. Afterwards, the plates were shaken and ZnPcOBP fluorescence was read in a microplate reader (λ_exc_ 620 nm; λ_em_ 675 nm).

The BCA protein kit was used for protein correlation after reading the ZnPcOBP fluorescence [42]. Thus, 150 µL from each well was transferred to a clear bottom 96 well plate, 150 µL of the BCA working reagent was added to each well, the plates were shaken for 30 s on a plate shaker, and they were finally incubated at 37 °C for 1 h. After incubation, the plates were cooled to room temperature and the absorbance of each well was measured at 562 nm with a microplate reader. The amount of protein was calculated by using the standard curve of BSA. Then the fluorescence of the wells was divided with correlated BSA concentration. Therefore, the cellular uptake of compounds was determined as ZnPcOBP fluorescence/protein amount (µg).

#### 4.4.3. In Vitro Dark- and Phototoxicity Assays

A quantity of 1.7 × 10^4^ cells was added to 96 wells plate and incubated at 37 °C for 24 h to achieve 80% confluence. Afterwards, the culture medium was removed and the previously-dissolved ZnPcOBP or suspended NPs or the equivalent amounts of DMA or PBS were added. After 24 h of incubation, the compounds were removed, the wells were washed 3 times with PBS and then fresh medium was added onto the cells. Dark controls were placed directly into the 37 °C incubator for 24 h, whilst light-treated plates were irradiated with 20, 30, and 40 J/cm^2^. For photodynamic treatments, the red light from an LED Par 64 Short V2 lamp (λ_em_ = 633 ± 9 nm; irradiance: 9.0 mW/cm^2^; Showtec, Kerkrade, The Netherlands) was used. After irradiation, the plates were placed into a 37 °C incubator for 24 h. Finally, the medium onto the cells was removed and the dark- and phototoxicity was evaluated using the 3-(4,5-dimethylthiazol-2-yl)-2,5-diphenyltetrazolium bromide (MTT) reduction assay [43].

## Figures and Tables

**Table 1:** Size and zeta potential of MSNP1, MSNP3, MSNP4 and MSNP5 nanoparticles.

Title 1	MSNP1	MSNP3	MSNP4	MSNP5
Size (nm)	126 ± 8	262 ± 15	285 ± 27	303 ± 26
Zeta Potential (mV)	−14 ± 4	−27 ± 1	−17 ± 5	−9 ± 7

## Supplementary Materials

Supplementary Materials are available online. **Figure S1**. SEM images of MSNP3 (a), MSNP4 (b) and MSNP5 (c). **Figure S2**. TEM images of MSNP3 (a), MSNP4 (b) and MSNP5 (c). **Figure S3**. EDS analysis graph of MSNP3 nanoparticles. **Figure S4**. Infrared spectra of ZnPcOBP (a), ITES (b), ITES-ZnPc (c), MSNP3 (d), MSNP4 (e) and MSNP5 (f). **Figure S5**. Top: UV-Vis absorption spectrum of 0.10–1.00 µM ZnPcOBP in tetrahydrofuran. Bottom: Calibration curve obtained from the absorbance data presented above at 680 nm. **Figure S6**. UV-Vis (a) and fluorescence (b; λ_exc_ = 620 nm) spectrum of 0.1–1 µM ZnPcOBP and 1 mg of MSNP3 in tetrahydrofuran. **Figure S7**. UV-Vis (a) and fluorescence (b; λ_exc_ = 620 nm) spectrum of 0.1–1 µM ZnPcOBP and 1 mg of MSNP4 in tetrahydrofuran. **Figure S8**. UV-Vis (a) and fluorescence (b; λ_exc_ = 620 nm) spectrum of 0.1–1 µM ZnPcOBP and 1 mg of MSNP5 in tetrahydrofuran. **Figure S9**. Singlet oxygen phosphorescence kinetic traces in toluene for optically-matched solutions of ZnPcOBP (green line) and phenalenone (PN; black line). λ_obs_ = 1275 nm. **Figure S10**. Singlet oxygen phosphorescence kinetic traces for MSNP3 (left) and MSNP4 (right) in the absence (black line) and presence (25 mM; green line) of NaN_3_ in methanol. λ_obs_ = 1275 nm. **Figure S11**. Right: One milliliter of MSNP-3 (left Eppendorf tube) and MSNP-4 (right Eppendorf tube) centrifuged for 10 min at 3000 rpm in pyridine. Left: UV-Vis spectra of the previous supernatants (black and red lines for MSNP-3 and MSNP-4 respectively). **Figure S12**. ASPC-1 (a–c), PANC-1 (d–f) and MIA PaCa-2 (g–i) cell viability (expressed in percentage) after incubation with: DMA (a,d,g) or ZnPcOBP (dissolved in DMA; b,e,h) and MSNP3 (suspended in DMA; c,f,i), at various ZnPcOBP concentrations and light fluences (20, 30 and 40 J/cm^2^). In the panels (a,d,g), equivalent amounts of DMA to those needed to reach the indicated concentrations of ZnPcOBP were added in the wells to assess the effect of medium dilution with DMA. ZnPcOBP concentrations are 0.1 µM (black), 0.5 µM (red), 1.0 µM (blue), and 5 µM (green). Cells incubated with complete growth medium, to which 100% viability was assigned, were used as controls to calculate the cell viability of the cells treated at the different conditions. Data are the mean ± SD from three independent experiments.
