# Supplementary material for: Selective Photokilling of Human Pancreatic Cancer Cells Using Cetuximab-Targeted Mesoporous Silica Nanoparticles for Delivery of Zinc Phthalocyanine
Source: Molecules. 2018 Oct 24;23(11):2749. doi: 10.3390/molecules23112749 (PMC6278564; doi:10.3390/molecules23112749)
Supplement: Supplementary file 1 [file molecules-23-02749-s001.pdf]

Supporting Information

Investigation of in vitro photodynamic therapy potential of tumor targeted zinc phthalocyanine loaded silica nanoparticles for pancreatic cells

Ozge Er [1], Suleyman Gokhan Colak [2], Kasim Ocakoglu [2,3], Mine Ince [3], Roger Bresolí-Obach [4], Margarita Mora [5], Maria Lluïsa Sagristá [5], Fatma Yurt [1,*] and Santi Nonell [4,*]

1 Department of Nuclear Applications, Institute of Nuclear Science, Ege University, Bornova, 35100, Izmir, Turkey.; ozgeer88@gmail.com, fatma.yurt.lambrecht@ege.edu.tr
2 Advanced Technology Research & Application Center, Mersin University, Ciftlikkoy Campus, TR-33343 Yenisehir, Mersin, Turkey; suleymangokhancolak@gmail.com
3 Mersin University, Department of Energy Systems Engineering Faculty of Tarsus Technology, Mersin 33480, Turkey; kasimocakoglu@gmail.com, mine.ince79@gmail.com
4 Institut Quimic de Sarria, Universitat Ramon Llull, Via Augusta 390, 08017, Barcelona, Spain; rogerbresolio@iqs.url.edu, santi.nonell@iqs.url.edu
5 Departament de Bioquímica i Biomedicina Molecular, Facultat de Biologia, Universitat de Barcelona, Avinguda Diagonal 645, E-08028 Barcelona, Spain; margarita.mora@ub.edu, mlsagrista@ub.edu

* Correspondence: fatma.yurt.lambrecht@ege.edu.tr; Tel:+90232-311-34-34, santi.nonell@iqs.url.edu; Tel.: +34-93-267-2000

Supporting Information

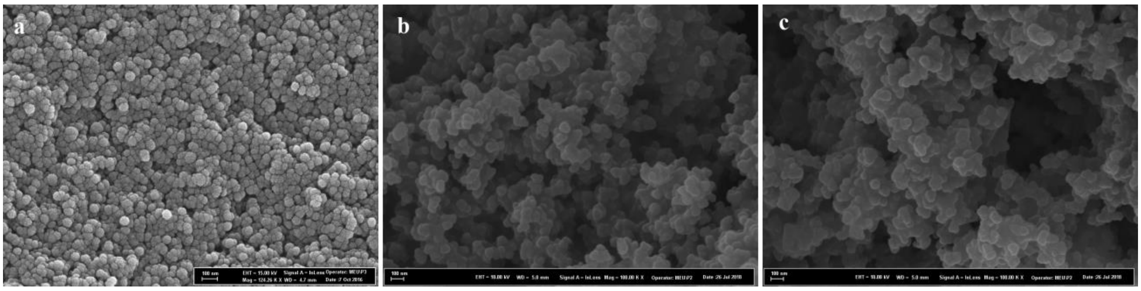

Figure S1. SEM images of MSNP3 (a), MSNP4 (b) and MSNP5 (c).

Supporting Information

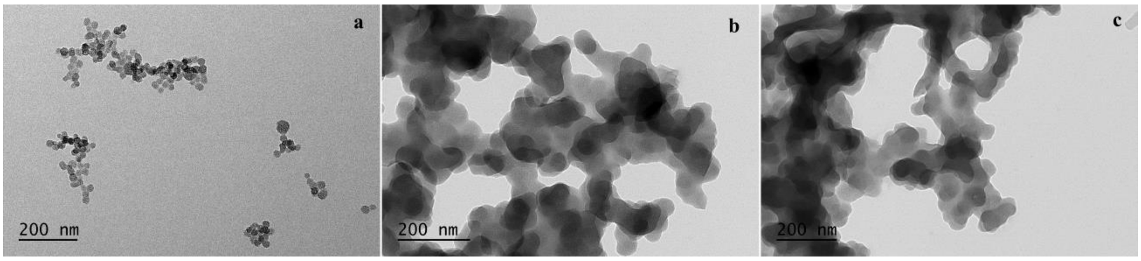

Figure S2. TEM images of MSNP3 (a), MSNP4 (b) and MSNP5 (c).

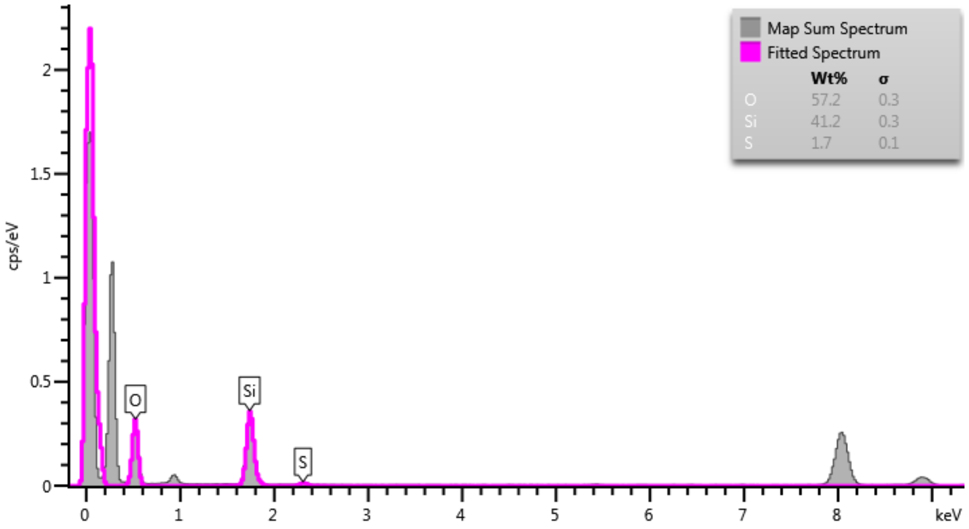

Figure S3. EDS analysis graph of MSNP3 nanoparticles.

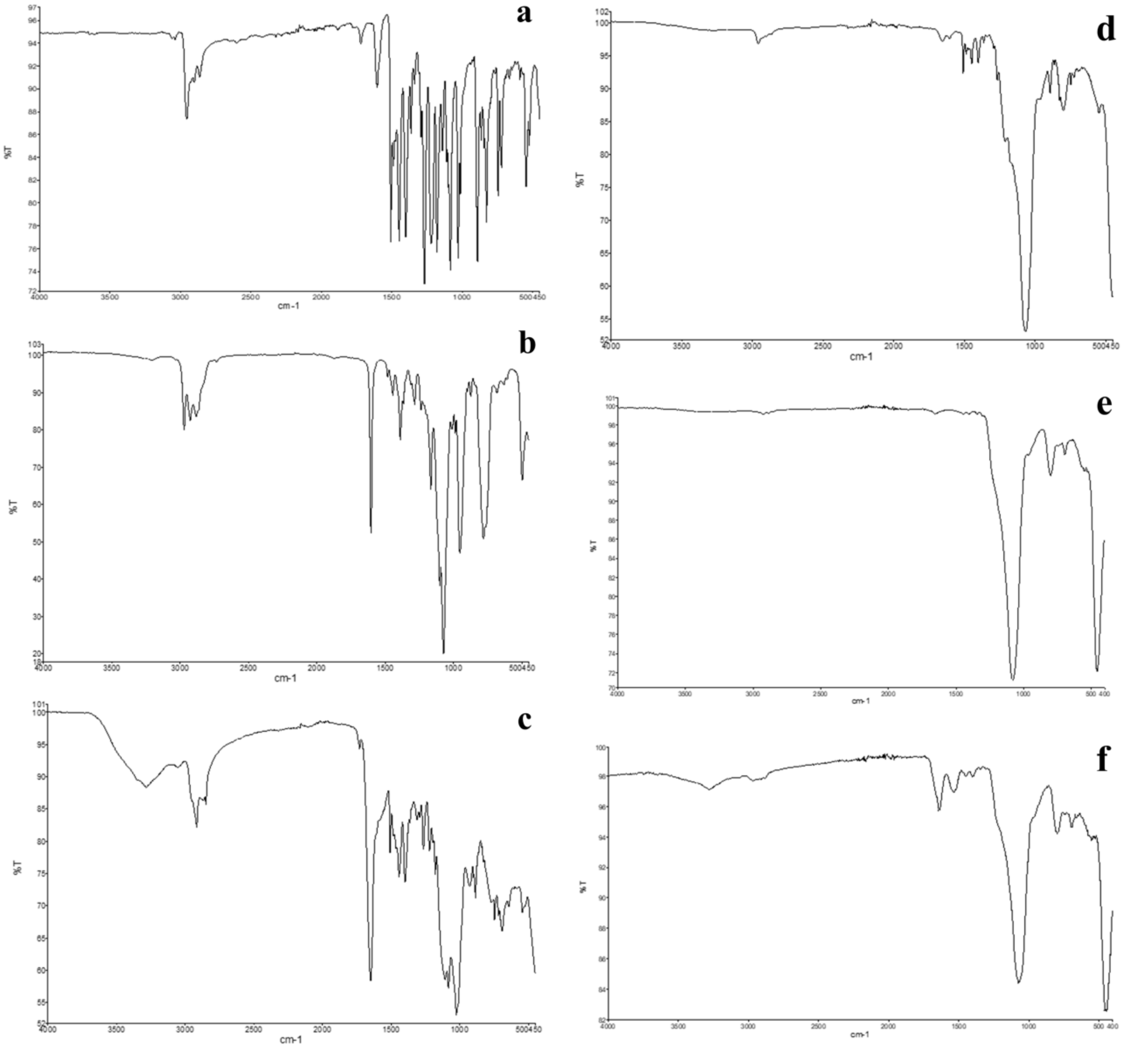

Figure S4. Infrared spectra of ZnPcOBP (a), ITES (b), ITES-ZnPc (c), MSNP3 (d), MSNP4 (e) and MSNP5 (f).

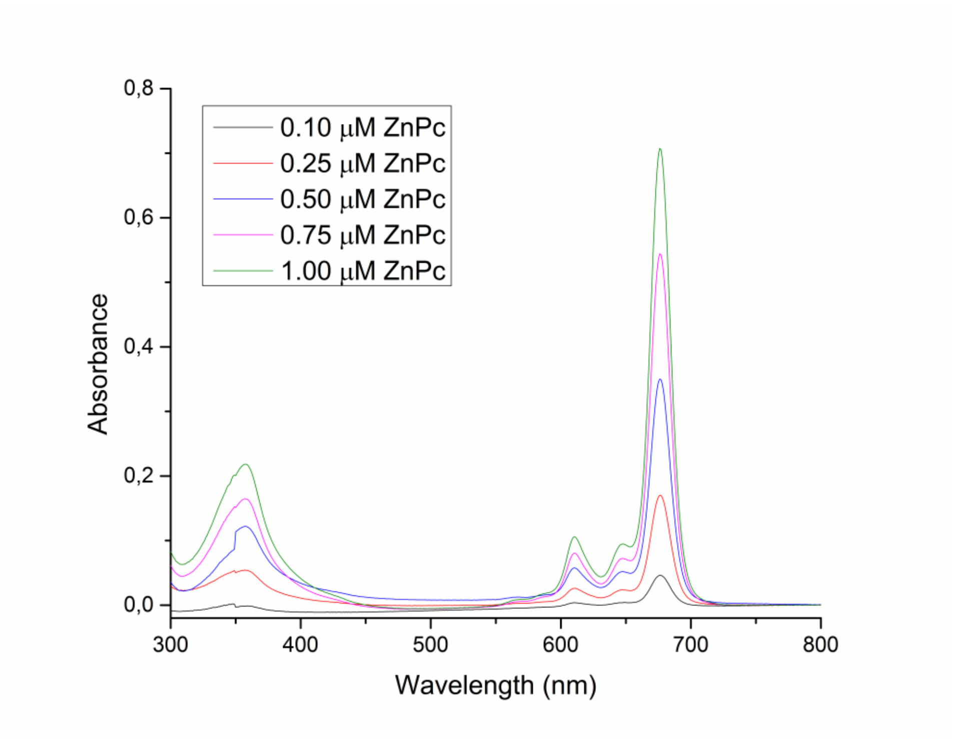

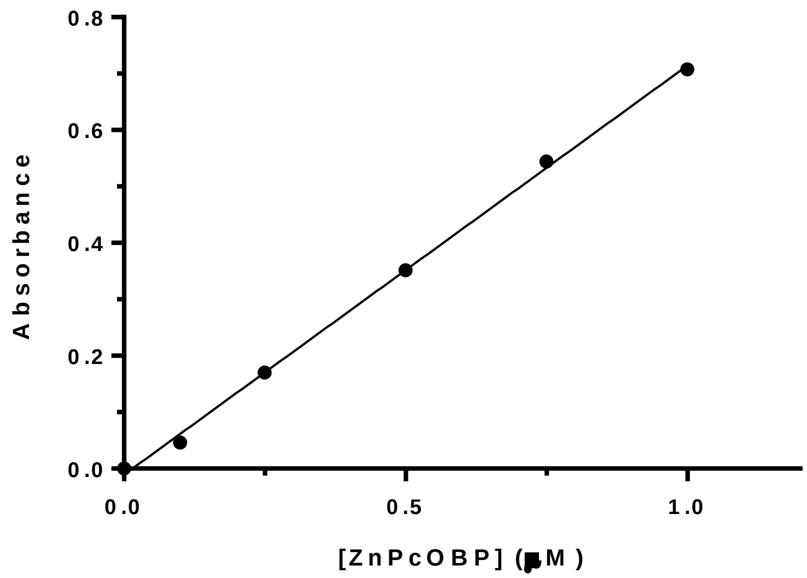

Figure S5. Top: UV-Vis absorption spectrum of 0.10-1.00 μM ZnPcOBP in tetrahydrofuran. Bottom: Calibration curve obtained from the absorbance data presented above at 680 nm.

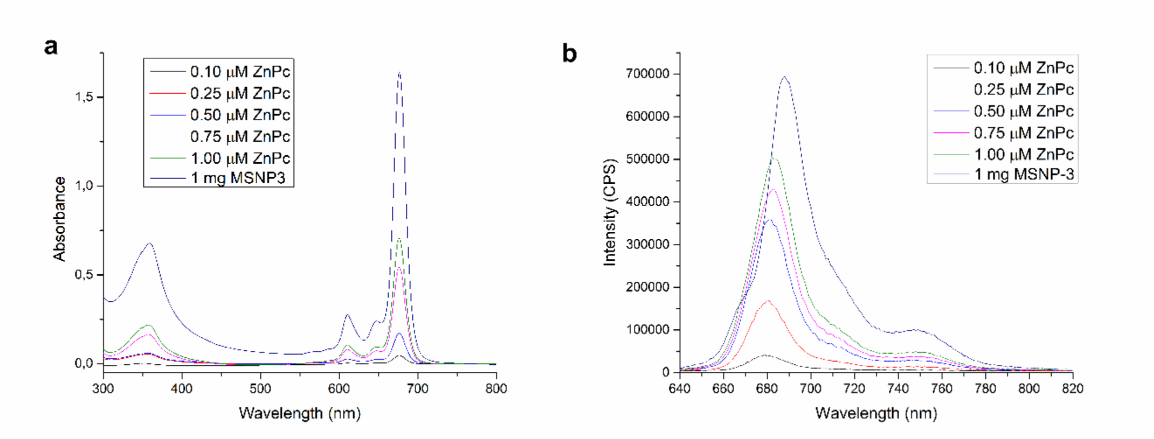

Figure S6. UV-Vis (a) and fluorescence (b; λ_{exc} = 620 nm) spectrum of 0.1-1 μM ZnPcOBP and 1 mg of MSNP3 in tetrahydrofuran.

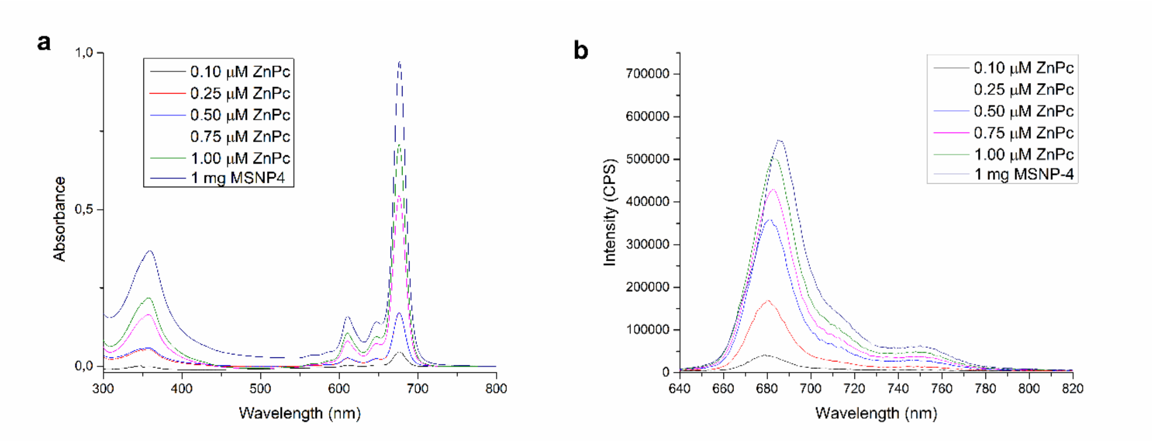

Figure S7. UV-Vis (a) and fluorescence (b; λ_{exc} = 620 nm) spectrum of 0.1-1 μM ZnPcOBP and 1 mg of MSNP4 in tetrahydrofuran.

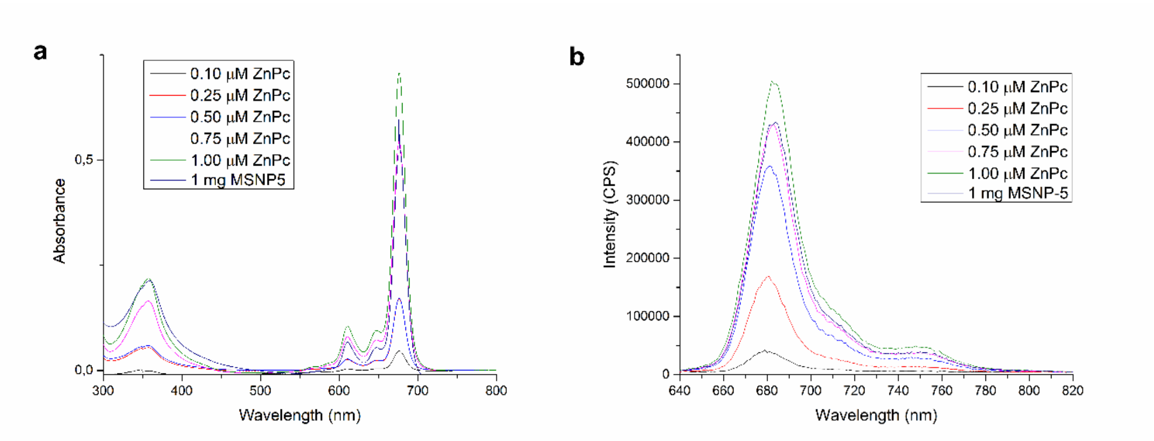

Figure S8. UV-Vis (a) and fluorescence (b; λ_{exc} = 620 nm) spectrum of 0.1-1 μM ZnPcOBP and 1 mg of MSNP5 in tetrahydrofuran.

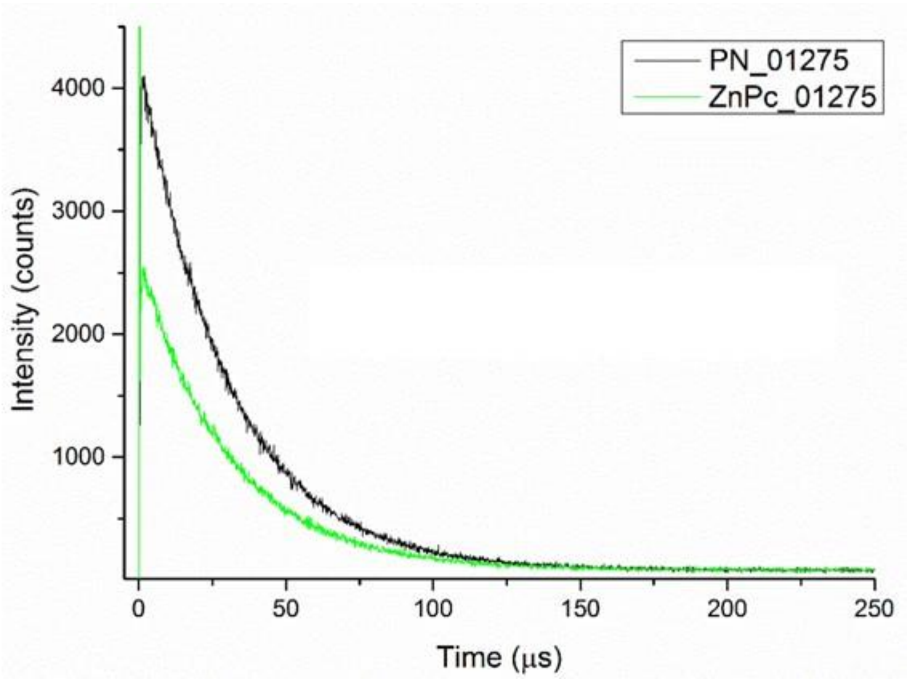

Figure S9. Singlet oxygen phosphorescence kinetic traces in toluene for optically-matched solutions of ZnPcOBP (green line) and phenalenone (PN; black line). λ_{obs} = 1275 nm.

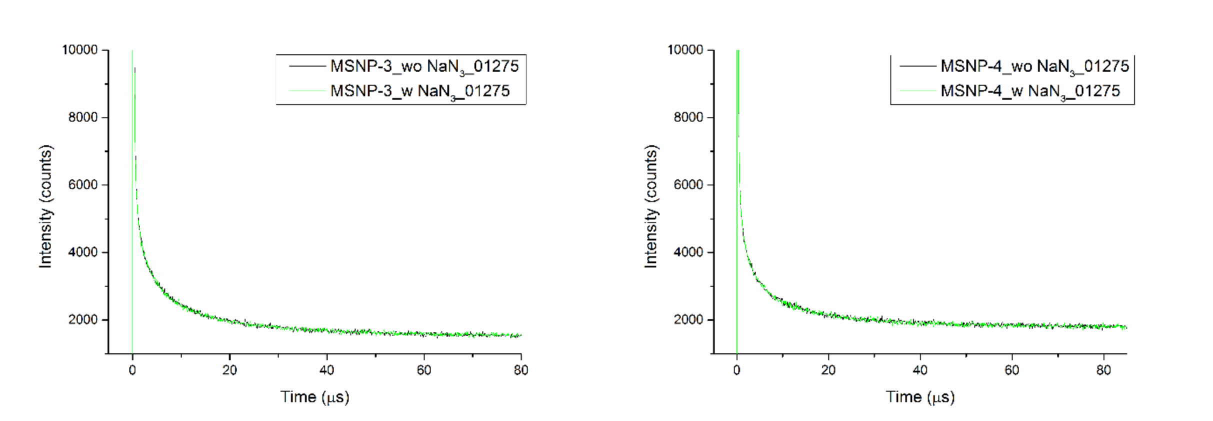

Figure S10. Singlet oxygen phosphorescence kinetic traces for MSNP3 (left) and MSNP4 (right) in the absence (black line) and presence (25 mM; green line) of NaN_3 in methanol. λ_{obs} = 1275 nm.

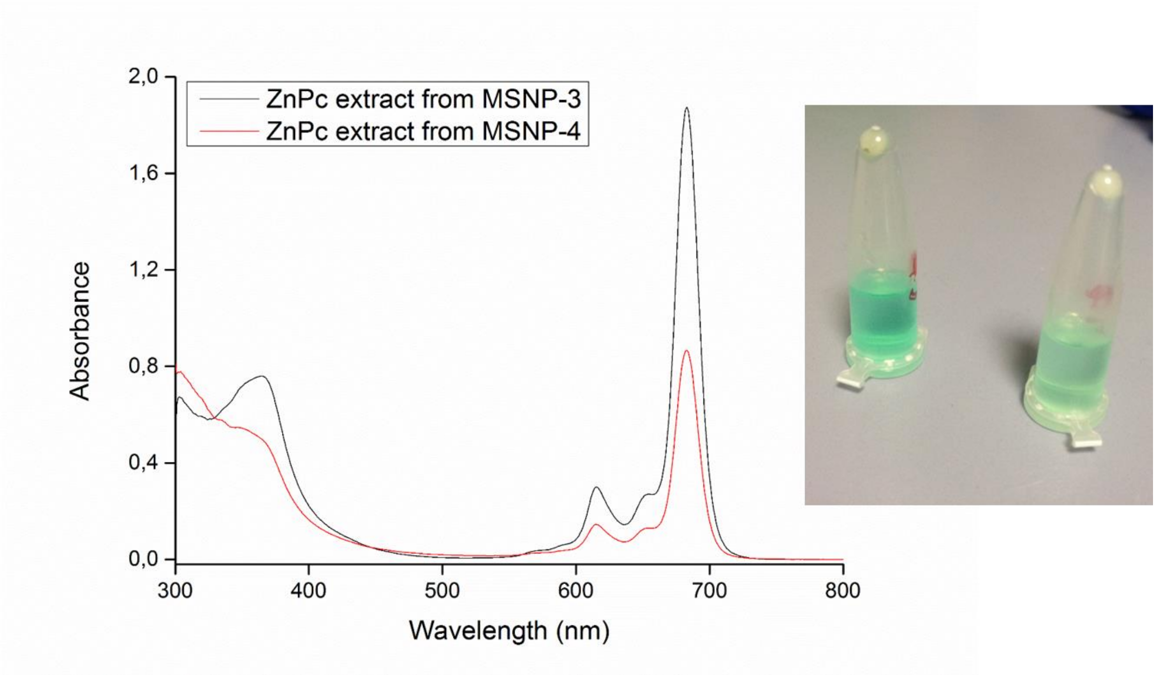

Figure S11. Right: One milliliter of MSNP-3 (left Eppendorf tube) and MSNP-4 (right Eppendorf tube) centrifuged for 10 min at 3000 rpm in pyridine. Left: UV-Vis spectra of the previous supernatants (black and red lines for MSNP-3 and MSNP-4 respectively).

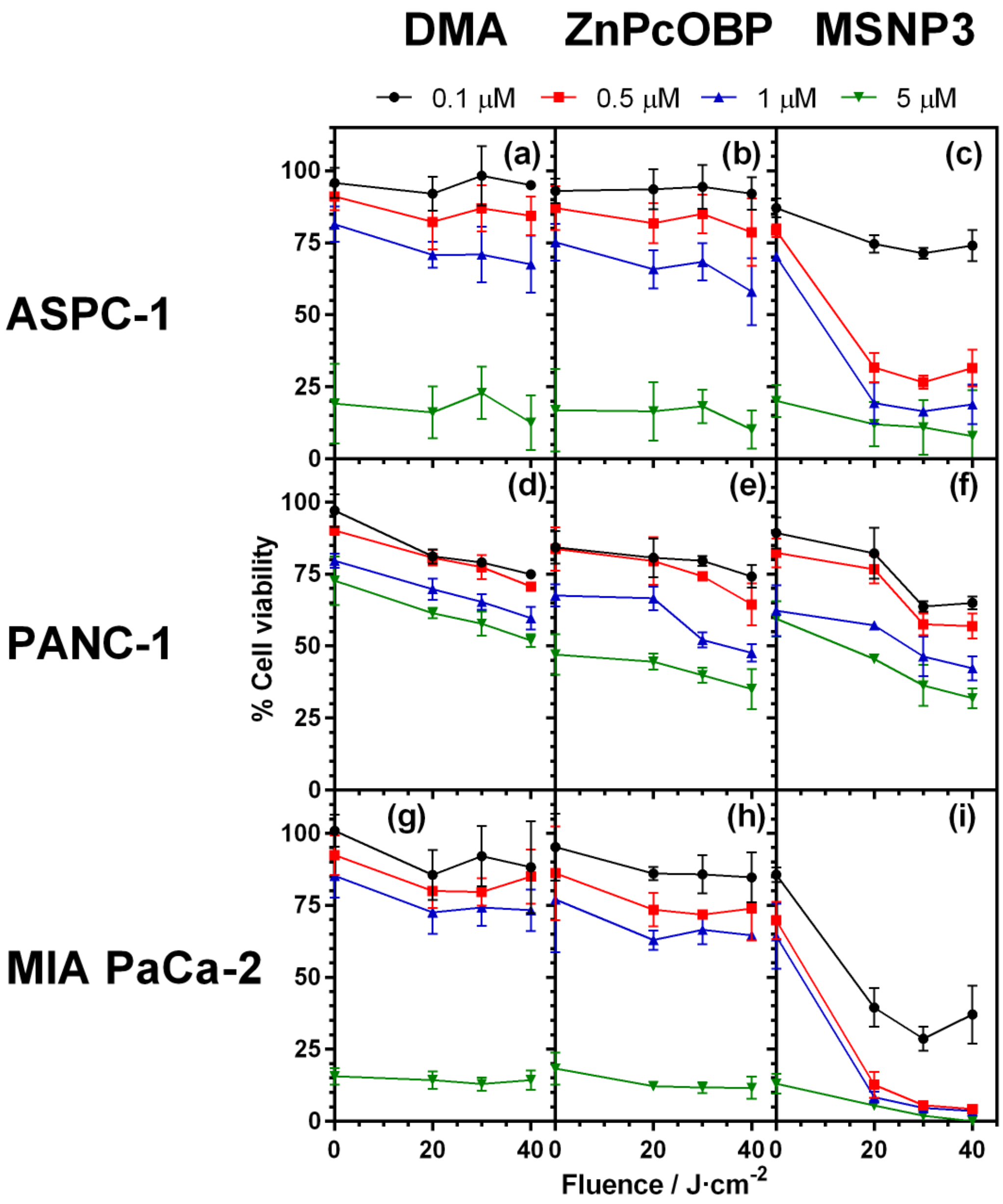

Figure S12. ASPC-1 (a-c), PANC-1 (d-f) and MIA PaCa-2 (g-i) cell viability (expressed in percentage) after incubation with: DMA (a,d,g) or ZnPcOBP (dissolved in DMA; b,e,h) and MSNP3 (suspended in DMA; c,f,i), at various ZnPcOBP concentrations and light fluences (20, 30 and 40 J/cm2). In the panels (a,d,g), equivalent amounts of DMA, to those needed to reach the indicated concentrations of ZnPcOBP were added in the wells to assess the effect of medium dilution with DMA. ZnPcOBP concentrations are 0.1 μM (black), 0.5 μM (red), 1.0 μM (blue) and 5 μM (green). Cells incubated with complete growth medium, to which 100% viability was assigned, were used as controls to calculate the cell viability of the cells treated at the different conditions. Data are the mean ± SD from three independent experiments.
